# Vegetation dynamics and soil nutrient availability in a temperate forest along altitudinal gradient of Nanda Devi Biosphere Reserve, Western Himalaya, India

**DOI:** 10.1371/journal.pone.0275051

**Published:** 2022-10-07

**Authors:** Ajay Maletha, Rakesh Kumar Maikhuri, Surendra Singh Bargali, Ajay Sharma, Vikram Singh Negi, Lakhpat Singh Rawat

**Affiliations:** 1 Amity Institute of Forestry and Wildlife, Amity University, Noida, Uttar Pradesh, India; 2 G.B. Pant National Institute of Himalayan Environment (NIHE), Garhwal Regional Centre, Srinagar Garhwal, Uttarakhand, India; 3 Department of Environmental Science, H.N.B. Garhwal University, Srinagar Garhwal, Uttarakhand, India; 4 Department of Botany, DSB Campus, Kumaun University, Nainital, Uttarakhand, India; 5 West Florida Research and Education Center, University of Florida, Milton, Florida, United States of America; 6 G.B. Pant National Institute of Himalayan Environment (NIHE), Kosi-Katarmal, Almora, Uttarakhand, India; University of Delhi, INDIA

## Abstract

This study examined forest structure, composition, and regeneration patterns of two sites, Tolma-Lata-Raini (TLR) and Bhyundar-Ghangaria (BG). Both sites are located within the temperate zone along the altitudinal gradient between 2,800 to 3,400 m asl of Nanda Devi Biosphere Reserve (NDBR) in the Western Himalayan Region of India. We recorded a total of 223 species of vascular plants (Angiosperm, Gymnosperm, and Pteridophytes) within the study area. Of the recorded species, plants within the family Rosaceae were dominant (17.69%), followed by Asteraceae (14.97%) and Ranunculaceae (12.93%). *Betula utilis* had the highest tree density (724 and 324 individuals ha^-1^) and species cover (44% and 36%) at both TLR and BG sites, followed by *Pinus wallichiana* (24%) and *Cedrus deodara* (15%), respectively. In BG site, 56% of tree species showed fair regeneration (i.e., seedling density > sapling density ≤ adult density), 22% good (i.e., seedling density > sapling density > adult tree density), 11% exhibited poor (i.e., species survived only in the sapling stage but not in the seedling stage), and the remaining (11%) indicated no regeneration. Comparatively, at TLR site, 40% of the tree species showed fair regeneration, 40% good, and the remaining 20% showed no regeneration. Across the two sites, species richness and diversity significantly decreased as the altitudinal gradient increased. Vegetation structure and soil properties also revealed differences between the southern and northern aspects. The baseline information generated in this study is helpful in designing effective conservation and management measures for these ecologically sensitive and important ecosystems. To effectively monitor changes in vegetation structure, species composition, and regeneration, we suggest that permanent vegetation plots with meteorological stations be established across the region for long-term monitoring of forest dynamics in response to the changing climate and anthropogenic pressures.

## Introduction

The Himalayan Region is among the most diverse ecosystems on earth, characterized by high species diversity, high endemism, and a variety of forest types. This high level of diversity is due to the existence of wide altitudinal gradient, complex topography, variety of soil types and climatic conditions, and unique geographical location of the Himalayan Region [1–6]. The Indian Himalayan Region (IHR), a section of the Himalayan Region in India, is a significant area of the country that covers only ~16.2% of the country’s total geographical area but accounts for nearly one-third of its forest cover [7]. It is a biodiversity hotspot harboring nearly 8,000 species of flowering plants (of which 25.3% are endemic [8]), a major carbon sink [9], a source of water for most of the major rivers in northern India, and livelihoods for millions of people. It is also one of the regions in the world that is most affected by climate change [10, 11]. Additionally, the forested landscape of the Indian Himalayan Region has been impacted by the local inhabitants who depend on its forest resources [7, 12], uncontrolled forest fires [13], shifting cultivation, agricultural expansion, and other land use changes [14]. Over the decades, these factors have led to forest degradation, deforestation, and biodiversity loss in the region [14]. Recognizing the ecological importance of the region and the natural and anthropogenic pressures it faces, India’s National Action Plan on Climate Change (NAPCC) considered this region highly vital to preserving the ecological security of India and enunciated the launch of a National Mission for Sustaining the Himalayan Eco-system (NMSHE) [15].

Being highly vulnerable to climatic and human disturbances, the forested landscape of the Indian Himalayan Region is likely to experience significant changes in its structure, composition, function, regeneration patterns, and altitudinal shifting of species’ native ranges [16–18]. The magnitude and nature of these changes will likely vary across the vast Indian Himalayan Region depending on forest type, topography, altitude, aspect, soils, and local socio-economic conditions. Unfortunately, we have a limited understanding of how the forest dynamics will alter in the face of the rapid climatic and anthropogenic changes that exist in the region. The forests and vegetation of the Indian Himalayan Region are inadequately studied, and, for many areas, even basic phytosociological information of the vegetation remains unavailable.

Several factors related to site and management affect vegetation. Altitude, among others, is one major factor that determines the structure and composition of vegetation in mountainous regions [19, 20]. Previous studies on vegetation in the Himalayas [21–29] and other parts of the world [30–35] observed a hump-shaped distribution of species along the altitudinal gradient. The hump-shaped distribution indicates that a higher species diversity exists at mid-level altitude, as opposed to low or high-level altitude in mountainous zones [36, 37]. Additionally, the population structure, characterized by the presence of an adequate number and proportion of seedlings, saplings and adults, is indicative of the healthy and balanced regeneration of a forest species [8, 27, 38–40]. However, the future composition of the forests depends on the potential regenerative status of tree species within a forest stand in space and time [18]. Currently, we have limited information on the regeneration status of the major Himalayan forest species and how they will respond to the changing climate and other factors.

Most the research work that has been carried out in the past involving the temperate zone of the Western Himalayan Region has focused on floristic diversity, plant community structure and composition, ethnobotanical surveys, ecosystem services, regeneration of trees, and biomass productivity and carbon storage [41–57], with only little attention paid to investigating vegetation dynamics in relation to human disturbances and edaphic conditions along the altitudinal gradients [51, 58–63]. In particular, the distribution of plant species in relation to soil nutrient availability and human disturbance in the region remains poorly investigated.

In this study we focused on characterising the forests and soil types of two select sites, Tolma-Lata-Raini (TLR) and Bhyundar-Ghangaria (BG). The sites are located in the Nanda Devi Biosphere Reserve (NDBR), which is a heavily protected area located in the western portion of the Indian Himalayan Region (Western Himalaya).

The TLR and BG sites in NDBR are representative of the unique ecological conditions of the region. Western Himalayan temperate dominant species in these two sites, such as *Cedrus deodara*, *Pinus wallichiana*, *Abies pindrow*, broad leaved *Quercus semicarpifolia*, and *Betula utilis*, are under severe anthropogenic pressure and are being exploited for fuelwood, fodder, timber, resin, and other non-timber forest products (NTFPs) [64]. The TLR site is located on an old trekking route that led to the core zone of NDBR, before the area was notified as a Biosphere Reserve in 1988 under the Man and the Biosphere (MAB) program of UNESCO. The BG site is heavily impacted by an influx of tourists who visit the Valley of Flower National Park (another core zone of NDBR) and the pilgrims who visit Hemkund Sahib (a sacred place for the adherents of Sikhism located within NDBR), in addition to the disturbances caused by the local inhabitants, businesses, and the associated economic activity. These disturbance factors have exerted an immense pressure on the local and regional biodiversity (flora and fauna), resulting in the decline of some of the dominant plant species populations. Additionally, widespread collection of NTFPs from some local species, such as *Ophiocordyceps sinensis*, *Morchella esculenta*,and lichen spp., over the decades has changed the vegetation dynamics of the region at different altitudinal zones, as different species exist at different altitudes.

We conducted this study with the following objectives: (1) characterise the vegetation structure and composition of the TLR and BG sites along an altitudinal gradient, (2) determine whether the vegetation structure, composition, and regeneration of the dominant plant species changes along the altitudinal gradient up to timberline ecotone, and (3) assess the nutrient availability of the soil and its relation to the vegetation characteristics. We hypothesised that vegetation and soil characteristics would significantly vary along the altitudinal gradient. Overall, this study investigated the change in vegetation and nutrient availability with an increasing altitudinal gradient in relation to the anthropogenic pressure being exerted by dependent communities that reside in the area. Findings of this study will provide insight into the regeneration dynamics of dominant species of the area and their implications for the future ecological trajectory of the regional vegetation communities. This scientific information is critical to the effective conservation and management planning of these sensitive and vital ecosystems.

## Materials and methods

### Ethics statement

This study was conducted after getting permission from Uttarakhand Forest Department (letter no- 280/5-6 dt. 30/06/2012). We followed all guidelines under wildlife protection act 1972 (amended 2006) and scientific research ethics.

### Study site

**Fig 1:**
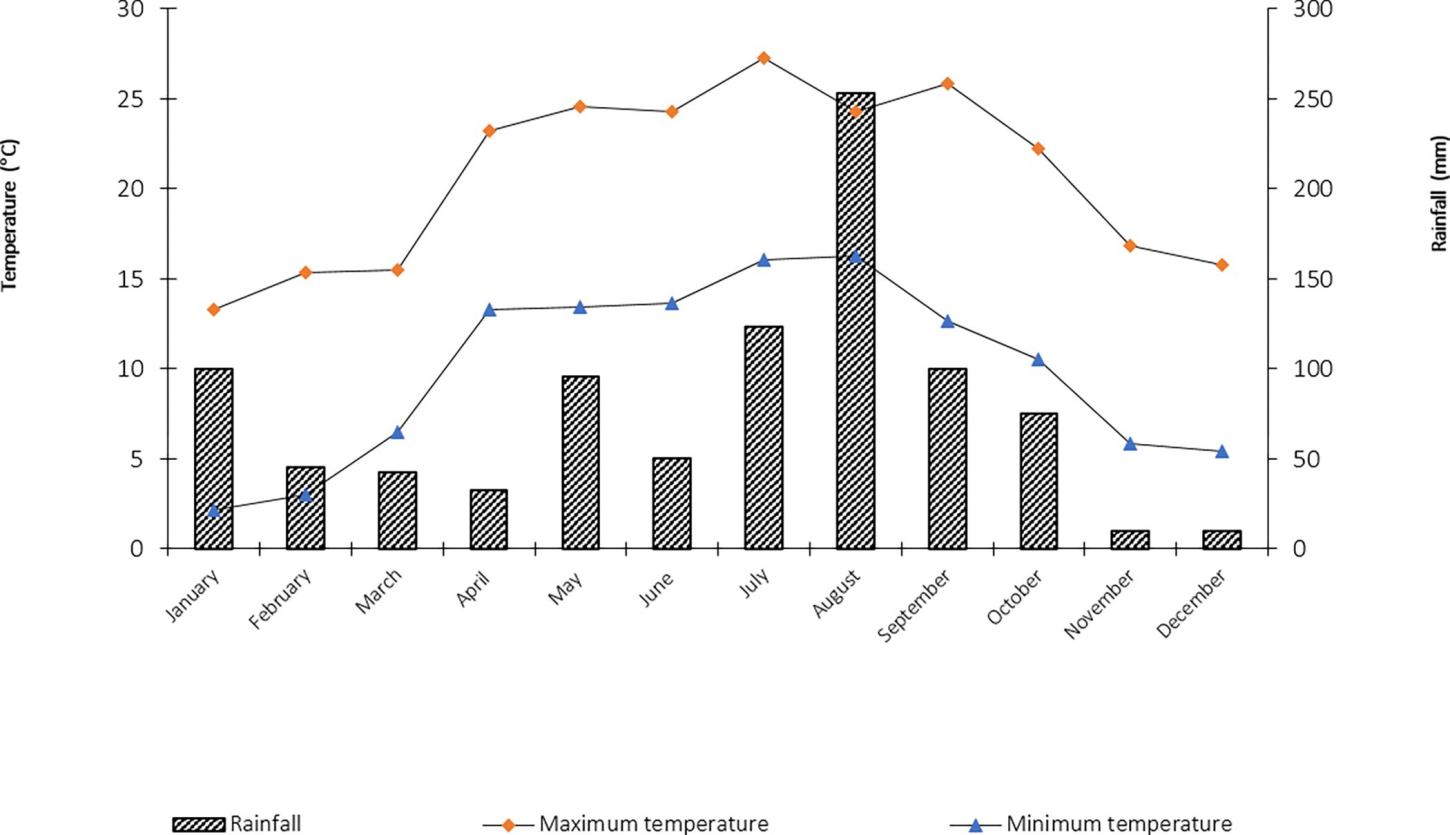
Climate data of the study area.

The study was conducted at two different sites within the Nanda Devi Biosphere Reserve (NDBR) nearby timberline ecotone viz.: (A) Bhyundar-Ghangaria site (BG site; The last human inhabitance on the way to famous Sikh shrine Hemkund Sahib and the Valley of Flowers National Park at an altitude of 3,050 m asl). (B) Tolma- Lata- Raini site (TLR site; small hamlets situated nearby the area spanning up to the core zone of Nanda Devi National Park). The sites cover area between 30°16’ N to 30° 41’ N and 79° 33’ E to 79° 44’ E with altitude ranges from 2,800 m to 3,400 m asl in the Western Himalayan Region. This biosphere reserve was recognized as a World Heritage Site in 1992 and was included in the UNESCO’s world network of Biosphere Reserves in 2004. The reserve covers an area of about 5,860 km^2^ with two core zones—the Nanda Devi National Park (624.62 sq. km) and the Valley of Flowers National Park (87.50 sq km). There are three seasons: summer (April-June), rainy season (June–September), and winter (October-March), and the average annual rainfall is 928.81 mm. About 47.8% of the annual rainfall occurs over a short period of two months (July-August) due to the strong influence of the monsoon. The maximum temperature ranges from 11 to 24°C and the minimum from 3 to 7.5°C (Fig 1). The altitudinal range of the biosphere reserve varies from 2,100 m asl to 7,817 m asl.

#### Vegetation sampling

Vegetation analysis was carried out by using the random sampling method at two sites along the timberline ecotone in NDBR. A total of 146 plots (73 in each site) of 10 m × 10 m were laid randomly in every 100 m altitude along the altitudinal gradient between 2,800 m asl to 3,400 m asl for enumeration of large number of plant species. Each plot (10×10 m) was further subdivided into 5 m × 5 m sub-plots for enumeration of shrubs and saplings and 1m × 1m sub-plots for seedlings and herb species [65]. The vegetation data was quantitatively analyzed for richness, abundance, density and frequency, following the methods of Curtis and McIntosh [66] and Misra [65]:

### Frequency


$$Frequency\;(\%)=\frac{Total\;number\;of\;quadrats\;in\;which\;species\;occurred}{Total\;number\;of\;quadrats\;studied}X\;100$$

### Density


$$Density=\frac{Total\;number\;of\;individuals\;of\;a\;species}{Total\;number\;of\;quadrats\;studied}$$

### Abundance


$$Abundance=\frac{Total\;number\;of\;individual\;of\;a\;species}{Total\;number\;of\;quadrats\;in\;which\;species\;occurred}$$

The importance value index (IVI) for the tree species was determined as the sum of three parameters viz.: relative density, relative frequency, and relative dominance, which were obtained by following the method used by Curtis [67]. Species diversity (H’) was calculated using the Shannon-Weaver index (1949) [68], where H = —∑ (Ni/N) log 2 (Ni/N). Simpson’s diversity index (Simpson, 1949) [69] was calculated as: D = 1-Cd, where D = Simpson’s diversity and Cd = Simpson’s concentration of dominance. The concentration of dominance is calculated as (∑Ni /N)^2^,where, Ni = total number of individuals of a species and N = total number of individuals of all species in the plot.

The regeneration status of dominant tree species was assessed based on the proportional distribution and density of individuals in the seedling, sapling and adult stages. The regeneration status of a tree was considered “**good**” when seedling density > sapling density > adult tree density, “**fair**” when seedling density > sapling density ≤ adult density, “**poor**” when the species survived in only the sapling stage but not in the seedling stage, “**none**” when species were not present in the sapling or seedling stages but were present as adult trees, and “**new**” when the adult stage of a species was absent but sapling and/or seedling stage(s) were present [70, 71].

#### Physiochemical properties of soil

A total of 56 composite soil samples were collected from 0–10 cm (surface layer), 10–20 cm (middle layer), and 20–30 cm (deeper layer) soil depths to determine the physiochemical characteristics of soil at different locations along the altitudinal gradient. The moisture percentage, water holding capacity (%), and texture of soil were determined as per the methods described by Misra (1968) [65]. Soil pH (1:2.5, soil: water) was measured using a dynamic digital pH meter. Soil organic carbon concentration (%) was determined by using Walkley and Black’s rapid titration method [72]. The total concentration of N was determined using the micro-Kjeldahl approach, while the concentrations of exchangeable phosphorus (P) and available potassium (K) were determined using the methods that were outlined by Jackson (1958) [73].

#### Statistical analysis

Different strata of vegetation (viz., tree sapling and seedling) matrices representing vegetation characteristics at different aspects (North and South aspects) were constructed using the Microsoft Excel for Windows (Ver. 10.0) software package for Principal Component Analysis (PCA) to determine the reciprocal effect of soil and vegetation. PCA is considered more effective if the cumulative percentage of variables approached is above 80 percent [74]. The scores of rotated component loadings (correlation coefficients) from the PCA output were used to determine the major vegetation components and their correlations. The rotated component loadings for the variables were determined using Varimax rotation (variance maximization); the idea of Varimax rotation is that each variable should load heavily on as few components as possible to make interpretation easier [75]. On each component, variables with loadings ≥0.05 were identified as significant variables and were used to describe the different components of the vegetation data structure. In addition, a t-test and correlation analysis were also performed for the species richness and diversity along the altitudinal gradient, showing significant correlation on vegetation characteristics up to the timberline zone of NDBR.

## Results

### Species richness and diversity

A total of 223 species of vascular plants (angiosperms, gymnosperms, and pteridophytes) were identified within the study area. These species belonged to 57 families and 174 genera. Of these, 46 families and 151 genera of angiosperms, 4 families and 8 genera of gymnosperms, and 7 families and 15 genera of pteridophytes were found at both study sites between 2,800–3,400 m asl (S1 Table; attached as supplementary material). Among the flowering plants (angiosperms), the proportional percentage of life forms were recorded as 4.6% trees, 18.77% shrubs, and the remaining 76.63% as herbs and forbs. Plants within the family Rosaceae were dominant (17.69%), followed by Asteraceae (14.97%), Ranunculaceae (12.93%), Lamiaceae (7.48%), and Poaceae (7.48%). (S2 Table; attached as supplementary material). The total species richness (tree and shrub) was found to be higher in the BG site when compared to the TLR site. Most of the tree and shrub species were common at both the sites, resulting in a 90% similarity in tree layer and 60% similarity in shrub layer. The Shannon-Weaver diversity index and Simpson diversity index values were slightly higher at the BG site (7.9 and 3.34), respectively. However, the shrub layer showed a higher Simpson’s diversity (0.92) at TLR site as compared to BG site (Table 1).

**Table 1:** Shannon and Weaver diversity index (H’) and Simpson’s diversity (D) for tree, sapling, seedling and shrub in BG and TLR sites.

Site	Parameters	Forest strata	Diversity	Dominant tree species
**BG site**	Shannon and Weaver diversity index (H’)	Tree	1.93	*Betula utilis*, *Cedrus deodar*, *Abies pindow*, *Taxus baccata*, *Acer caesium*, *Salix sikkimensis*, *Rhododendron campanulatum*
		Sapling	1.99	
		Seedling	1.99	
		Shrub	1.99	
		**Total**	**7.9**	
	Simpson’s diversity(D)	Tree	0.82	
		Sapling	0.84	
		Seedling	0.85	
		Shrub	0.83	
		**Total**	**3.34**	
**TLR site**	Shannon and Weaver diversity index (H’)	Tree	1.65	*Betula utilis*, *Abies pindrow*, *Pinus wallichiana*, *Salix sikkimensis*, *Cedrus deodar*, *Populus ciliata*, *Picea smithiana*, *Acer caesium*
		Sapling	1.87	
		Seedling	1.77	
		Shrub	2.55	
		**Total**	**7.84**	
	Simpson’s diversity (D)	Tree	0.74	
		Sapling	0.80	
		Seedling	0.78	
		Shrub	0.92	
		**Total**	**3.24**	

### Population structure

The total tree density (1,632 trees ha^-1^) and basal area (67.39 m^2^ ha^-1^) was higher in the TLR site as compared to the BG site. Among the total tree density, the highest value (724 trees ha^-1^) was recorded for *Betula utilis*, with the highest IVI (114.35) value occurring at the same site. *Cedrus deodara* was found to be an important co-dominant tree species (IVI 63.43) at the BG site. *Pinus wallichiana* (IVI 70.84) and *Abies pindrow* (IVI 58.42) were found to be important co-dominant tree species at the TLR site, respectively (Table 2).

**Table 2:** Phytosociological parameters of tree species around temperate zone of Nanda Devi Biosphere Reserve, Western Himalaya.

Tree species	BG site			TLR site		
	Density trees ha^-1^	TBC m^2^ ha^-1^	IVI	Density trees ha^-1^	TBC m^2^ ha^-1^	IVI
*Betula utilis*	368	6.7	85.68	724	12.8	114.35
*Abies pindrow*	128	4.8	48.70	248	41.3	58.42
*Cedrus deodara*	156	8.5	63.43	44	0.45	7.20
*Pinus wallichiana*	-	-	-	384	11.9	70.83
*Taxus baccata*	132	3.2	38.93	60	0.19	11.51
*Acer caesium*	80	0.1	22.64	20	0.04	5.84
*Sorbus foliolosa*	36	0.2	10.23	-	-	-
*Rhododendron campanulatum*	44	0.3	12.33	-	-	-
*Salix sikkimensis*	20	0.3	5.690	68	0.2	12.63
*Populus ciliate*	-	-	-	60	0.36	13.73
*Picea smithiana*	-	-	-	24	0.11	5.44
*Prunus cornuta*	52	1.0	12.33	-	-	-
**Total**	**1,016**	**25.18**	**300**	**1,632**	**67.39**	**300**

In the shrub layer, *Ribes alpestre* (396 individuals ha^-1^), *Origanum vulgure* (332 individuals ha^-1^), *Spiraea bella* (224 individuals ha^-1^), *Polygonum polystachyum* (176 individuals ha^-1^), and *Rosa webbiana* (132 individuals ha^-1^) were found in a higher density at the BG site. Comparatively, at the TLR site, the highest density was represented by *Berberis jaeschkeana* (228 individuals ha^-1^), followed by *Salix sikkimensis* (176 individuals ha^-1^), *Indigofera heterantha* (152 individuals ha^-1^), and *Sorbus microphylla* (120 individuals ha^-1^) (Table 3). In the herbaceous layer at the BG site, the dominant species were found in the following sequence: *Fragaria nubicola* (844 individuals ha^-1^)> *Oxalis corniculata* (812 individuals ha^-1^)> *Geranium wallichianum* (780 individuals ha^-1^*)> Anaphalis triplinervis* (736 individuals ha^-1^)> *Impatiens sulcata* (364 individuals ha^-1^). The other co-dominant species included *Potentilla atrisanguinea*, *Fragaria nubicola*, *Anemone obtusifolia*, *Ligularia amplexicaulis*, *Origanum vulgare*, *Senecio graciliflorus*, etc. However, the TLR site exhibited the highest density of *Geranium himalayense* (1384 individuals ha^-1^), followed by *Oxalis corcunata* (792 individuals ha^-1^), and *Fragaria nubicola* (756 individuals ha^-1^). Within the TLR site, the species with the lowest density were identified as *Angelica glauca* (48 individuals ha^-1^), *Bergenia ciliata* (112 individuals ha^-1^), and *Malaxis muscifera* (140 individuals ha^-1^). Some other prominent species that were found in the area were *Berginia starchy*, *Bistorta affinis*, *Geum elatum*, *Impatiens devendrae*, *Taraxacum officinale*, *Origanum vulgare*, etc. (Table 4).

**Table 3:** Phytosociological parameters of shrub species around temperate zone of Nanda Devi Biosphere Reserve, Western Himalaya.

Species	BG site			TLR site		
	Density Ind. ha^-1^	A/F ratio	IVI	Density Ind. ha^-1^	A/F ratio	IVI
*Astragalus chlorostachys*	-	-	-	108	0.421	11.96
*Berberis aristate*	-	-	-	60	0.234	8.81
*Berberis jaeschkeana*	72	0.18	14.36	228	0.291	23.49
*Colquhounia coccinea*	-	-	-	88	0.22	11.87
*Elsholtzia fruticose*	-	-	-	88	0.22	11.87
*Fren spp*	80	1.25	9.5	-	-	-
*Indigofera heterantha*	-	-	-	152	0.38	16.07
*Lonicera microphylla*	64	0.44	10.15	-	-	-
*Origanum vulgure*	332	0.32	38.88	-	-	-
*Prinspia utilis*	64	0.25	11.96	88	0.22	11.87
*Polygonum polystachyum*	176	0.13	22.64	-	-	-
*Ribies alpester*	396	0.31	35.39	92	0.159	13.35
*Rosa microphylla*	80	0.1	14.59	112	0.143	15.886
*Rosa webbiana*	132	0.23	14.58	-	-	-
*Rosa serica*	-	-	-	80	0.139	12.56
*Rubus niveus*	-	-	-	56	0.072	12.21
*Sorbus microphylla*	112	0.14	11.94	120	0.092	18.84
*Sorbaria tomentosa*	32	0.13	9.62	76	0.132	12.30
*Spiraea bella*	224	0.22	7.46	-	-	-
*salix lindleyana*	-	-	-	176	0.306	18.86
**Total**	**1364**	**3.35**	**200**	**1524**	**3.029**	**200**

**Table 4:** Density (Ind.m^-2^) of herbaceous species at BG and TLR sites around the temperate region of Nanda Devi Biosphere Reserve, Western Himalaya.

Plant species name	BG site	TLR site
*Anaphalis triplinervis*	-	736
*Anaphalis contorta*	130	-
*Anaphalis royleana*	136	-
*Anemone obtustifolia*	-	236
*Angelica glauca*	-	92
*Arisaema intermedium*	60	30
*Artemisia nilagirica*	320	-
*Aster thomsonii*	22	-
*Bistorta affinis*	-	540
*Bistorta vaccinifolia*	680	490
*Carex nubigena*	100	-
*Calquhounia coccinea*	80	128
*Cardamine impatiens*	122	-
*Carum carvi*	-	60
*Circaea alpine*	10	20
*Clematis montana*	-	52
*Clinopodium umbrosum*	106	-
*Codonopsis rotundifolia*	6	-
*Cremanthodium arnicoides*	56	-
*Cyananthus linifolium*	4	-
*Dactylorhiza hatagirea*	-	140
*Elsholtzia eriostachya*	50	-
*Epilobium latifolium*	8	-
*Eritrichium canum*	6	150
*Filipendula vestita*	6	-
*Fragaria nubicola*	170	844
*Fragaria vesca*	138	-
*Fritillaria royeli*	66	-
*Gentiana albicalyx*	35	-
*Gentiana phyllocalyx*	50	-
*Geranium wallichianum*	440	780
*Gerbera pusilla*	-	250
*Geum elatum*	10	-
*Impatiens sulcata*	-	364
*Inula grandiflora*	90	140
*Iris kemaonensis*	-	132
*Leucas lantana*	40	136
*Ligularia amplexicaulis*	-	196
*Meconopsis aculeate*	-	140
*Megacarpaea polyandra*	48	36
*Myosotis sylvatica*	90	-
*Oxalis corniculate*	-	812
*Oxitropis mirophylla*	-	-
*Potentilla fulgens*	94	-
*Pedicularis bicornuta*	66	-
*Phlomis bractiosa*	70	108
*Picrorhiza kurrooa*	-	70
*Pleurospermum candollii*	4	-
*Podophyllum haxendrum*	-	8
*Polygonum capitatum*	30	160
*Potentilla atrosangiunea*	40	292
*Potentilla microphylla*	-	8
*Ranunculus hyrtellus*	30	-
*Rheam austrail*	-	20
*Rheum webbianum*	60	96
*Rumax nepalensis*	6	8
*Senecio edgewarthi*	12	-
*Senecio setisperma*	180	-
*Salvia hians*	40	-
*Saussaurea auriculata*	106	192
*Saussaurea costus*	-	96
*Sedum imbricatum*	6	-
*Selinum vaginatum*	-	60
*Selinum wallichianum*	-	64
*Senecio graciliflorus*	68	200
*Silene indica*	-	-
*Silene vulgaris*	60	-
*Stellaria himalayensis*	130	-
*Thalictrum minus*	76	-
*Thalictrum elegans*	-	124
*Thalictrum foliolosum*	110	320
*Verbascum Thapsus*	10	-
*Viola biflora*	90	-

### Regeneration status

All 12 tree species were found along an altitudinal gradient between 2,800–3,400 m asl at both buffer areas of NDBR. Among all the tree species, 56% showed fair regeneration, 22% showed good regeneration, 11% exhibited poor, and remaining (11%) indicated no regeneration at the BG site. At the TLR site, 40% of species showed fair regeneration, 40% showed good regeneration, and the remaining 20% exhibited no regeneration. In the BG site, two species viz., *Salix sikkimensis* and *Rhododendron campanulatum*, showed good regeneration, as both the species were represented by a significant number of seedlings and saplings. However, about five plant species showed fair regeneration, including *Betula utilis*, *Abies pindrow*, *Taxus baccata*, *Cedrus deodara*, and *Sorbus tomentosa*.Species, such as *Acer caesium*, showed poor regeneration, and *Prunus cornuta* exhibited no regeneration. In the TLR site, ~four species (viz., *Salix sikkimensis*, *Populus ciliata*, *Taxus baccata* and *Cedus deodara*) showed good regeneration (Table 5). The highest seedling density was recorded for *R*. *campanulatum* (320 sapling ha^-1^) and *Betula utilis* (232 sapling ha^-1^) at the BG site. At the TLR site, the species with the highest densities were *Betula utilis* (544 sapling ha^-1^) and *Salix sikkimensis* (268 sapling ha^-1^). *Abies pindrow* was another associated species exhibiting a high sapling density at both sites (168 sapling ha^-1^ and 244 sapling ha^-1^ each site). In the seedling stratum, the maximum total density was recorded at the TLR site (2328 seedling ha^-1^) as compared to the BG site (2032 seedling ha^-1^). Among the species, higher seedling density was recorded for *Betula utilis* at both sites (920 seedling ha^-1^ and 456 seedling ha^-1^ each site), followed by *Rhododendron campanulatum* (428 seedling ha^-1^) and *Abies pindrow* (364 seedling ha^-1^) (Table 5).

**Table 5:** Regeneration status of trees species around BG and TLR sites, Nanda Devi Biosphere Reserve, Western Himalaya.

Dominant tree species	BG site				TLR site			
	Tree	Sapling	Seedling	Status	Tree	Sapling	Seedling	Status
*Betula utilis*	484	232	456	F	852	544	920	F
*Abies pindrow*	192	168	340	F	392	244	364	F
*Salix sikkimensis*	76	148	196	G	68	268	344	G
*Pinus wallichiana*	-	-	-	SA	464	100	200	F
*Acer caesium*	80	156	104	P	20	68	-	NR
*Populus ciliata*	-	-	-	SA	60	80	100	G
*Taxus baccata*	196	164	300	F	60	68	176	G
*Picea smithiana*	-	-	-	SA	24	-	-	NR
*Cedus deodara*	180	60	100	F	44	96	104	G
*Rhododendron campanulatum*	172	320	428	G	108	56	120	F
*Sorbus tomentosa*	36	140	108	F	-	-	-	SA
*Prunus cornuta*	52	-	-	NR	-	-	-	SA
**Total**	**1468**	**1388**	**2032**		**2092**	**1524**	**2328**	

Note: F = Fair regeneration; G = Good regeneration; P = Poor regeneration; NR = No regeneration; SA = Species absent.

### Soil characteristics

Soil water holding capacity (49.94±5.63%) and moisture content (60.43±9.71%) was found to be higher in the northern aspect as compared to southern aspect at the TLR site. However, the BG site also exhibited a higher water holding capacity (33.50±4.54%) in the northern aspect, while soil moisture was higher (63.77±4.77%) in the southern aspect. The TLR site revealed the highest bulk density for the southern aspect (0.91±0.10 g cm^3^), as compared to northern aspect, while the BG site ranged between 1.03 g cm^3^ to 1.06 g cm^3^ in the southern and northern aspects, respectively. As far as soil texture is concerned, sand was the prime constituent of the soil in both aspects and sites. However, a decreasing trend was observed in silt proportion with increasing altitude. The soil pH ranged between 6.54 to 6.77 and 6.28 to 6.94 in the TLR and BG sites, respectively. The availability of phosphorus (0.11±0.01%), potassium (1.32±0.56%), total organic carbon (1.04±0.30%), and organic matter (1.79±0.52%) was found to be higher in the southern aspect. The nitrogen concentration (0.170±0.07%) was recorded higher in the northern aspect of the TLR site, whereas the phosphorus concentration revealed a similar value (0.09 ±0.01%) for both aspects, while the potassium concentration (1.33±0.27%) was recorded higher in the northern aspect. The total nitrogen value (0.218±0.06%), total organic carbon (1.06±0.25%), and organic matter (1.82±0.44%) were the highest in the southern aspect at the BG site (Table 6).

**Table 6:** Physiochemical properties of soil in TLR and BG sites.

Parameter	BG site		TLR site	
	N aspect	South aspect	N aspect	South aspect
Sand (%)	63.98±8.27	46.88±2.70	60.11±4.13	64.99±3.95
Silt (%)	50.4±5.45	34.65±2.71	33.01±4.11	27.20±3.16
Clay (%)	0.88±0.06	0.87±0.12	6.88±2.80	7.82±1.41
Moisture (%)	57.41±3.27	63.77±4.77	60.43±9.71	46.49±3.85
WHC (%)	33.50±4.54	28.70±3.35	49.94±5.63	40.58±6.91
bD (g cm^-3^)	1.06±1.71	1.03±3.29	0.85±0.13	0.91±0.10
pH	6.94±0.15	6.28±0.22	6.77±0.30	6.54±0.37
P (%)	0.09±0.01	0.09±0.02	0.09±0.02	0.11±0.01
K (%)	1.33±0.27	1.23±0.69	1.32±0.56	1.47±0.72
N (%)	0.141±0.05	0.218±0.06	0.170±0.07	0.105±0.02
SOC (%)	0.89±0.11	1.06±0.25	0.94±0.15	1.04±0.30
OM (%)	1.54±0.20	1.82±0.44	1.62±0.25	1.79±0.52

WHC = Water holding capacity; bD = Bulk density; pH = Potential of hydrogen; P = Phosphorus; K = Potassium; N = Nitrogen; SOC = Soil organic carbon; OM = Organic matter

### Species composition and site similarity

**Fig 2:**
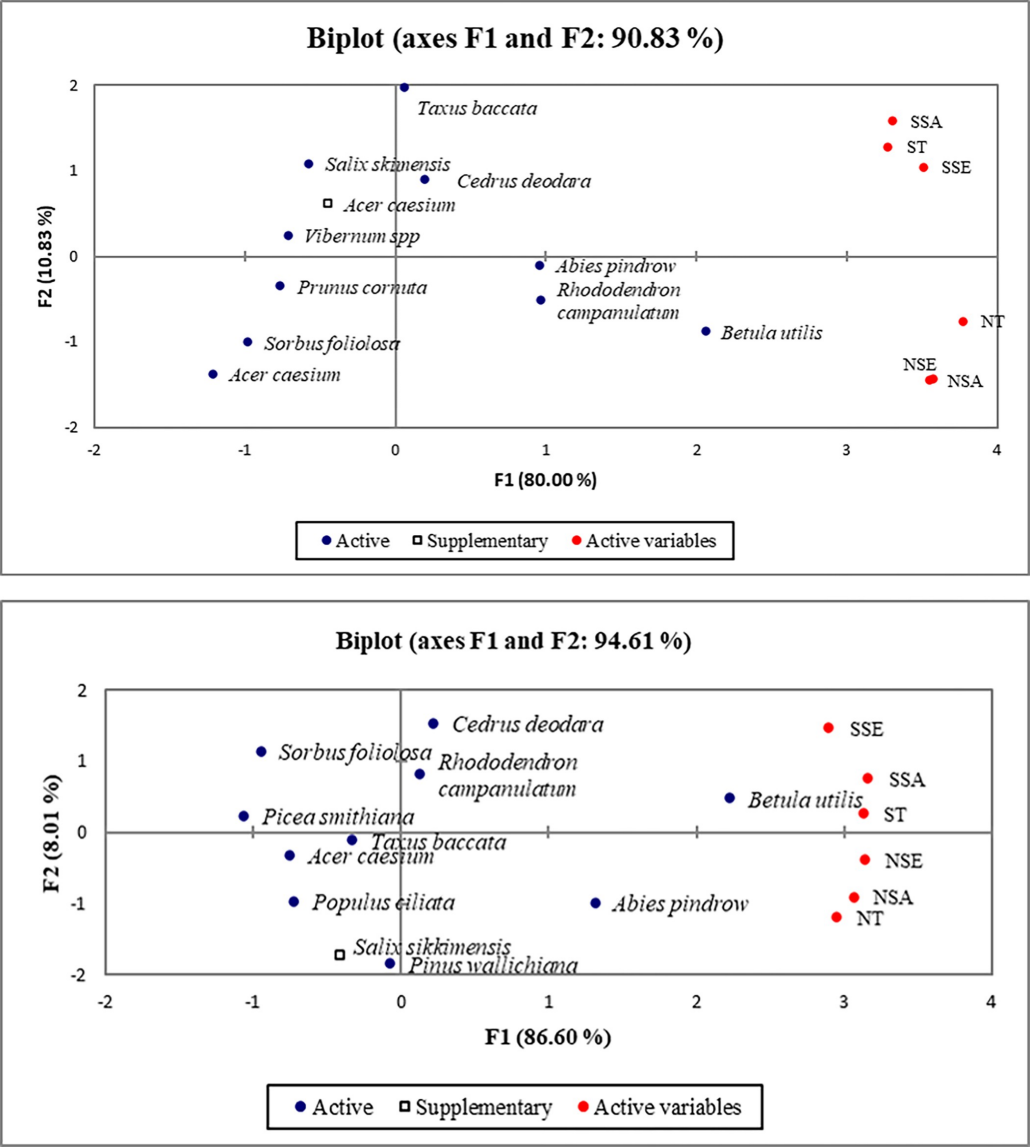
Principle component analysis (PCA) on the basis of frequency quality indicator at BG and TLR sites.

In the correlation analysis, we found significant positive correlation among the variables in the BG and TLR sites. The PCA ordination of the BG and TLR sites showed no relation of *Acer caesium* and *Salix sikkimensis* with any variable of the F1 and F2, which is indicated as a supplementary variable, respectively (Fig 2).

In the context of frequency, the occurrence of individuals in the seedling, sapling, and tree stages on the southern aspect was highly correlated with individuals in the seedling, sapling and tree stages on the northern aspect of both sites within the Reserve (significant at p≤0.05) (Tables 7 and 8). This analysis provides a clearer picture of the complex interrelationships among the different vegetation layers.

**Table 7:** Correlation matrix of floristic composition on the basis of frequency indicator at BG site, Nanda Devi Biosphere Reserve, Western Himalaya.

Variables	NT	NSA	NSE	ST	SSA	SSE
NT	**1**	**0.932** *	**0.943** *	**0.786** *	**0.727** *	**0.766** *
NSA		**1**	**0.935** *	0.578	**0.650** *	**0.744** *
NSE			**1**	**0.667** *	0.610	**0.712** *
ST				**1**	**0.722** *	**0.757** *
SSA					**1**	**0.843** *
SSE						**1**

*. Correlation is significant at the 0.05 level (2-tailed).

NT = North Tree; NSA = North Sapling; NSE = North seedling; ST = South Tree; SSA = South Sapling; SSE = South seedling

**Table 8:** Correlation matrix of floristic composition on the basis of frequency indicator at TLR site, Nanda Devi Biosphere Reserve, Western Himalaya.

Variables	NT	NSA	NSE	ST	SSA	SSE
NT	**1**	**0.897** *	**0.866** *	**0.802** *	**0.809** *	**0.636** *
NSA		**1**	**0.921** *	**0.861** *	**0.818** *	**0.707** *
NSE			**1**	**0.881** *	**0.863** *	**0.798** *
ST				**1**	**0.935** *	**0.831** *
SSA					**1**	**0.946** *
SSE						**1**

* Correlation is significant at the 0.05 level (2-tailed).

NT = North Tree; NSA = North Sapling; NSE = North seedling; ST = South Tree; SSA = South Sapling; SSE = South seedling

### Relationship between species richness and species diversity

The t-test value for tree and herbaceous species density along the altitudinal gradient showed significant variation (p<0.05). However, there were no significant differences in shrub species along the altitudinal gradient (P< 0.05). On the other hand, the correlation between vegetational parameters vs soil parameters (total nitrogen, phosphorus, potassium, and organic carbon) were also positive (p< 0.05). Species richness significantly decreased with the increasing altitudinal gradient (R^2^ = 0.826, p< 0.003). Simpson’s Index of Dominance and the altitude showed a positive quadrate relation (R^2^ = 0.326, p< 0.066). Both forest sites revealed declining trends for the Shannon-Weaver Diversity Index (R^2^ = 0.434, p< 0.026) and the Shannon Index of species evenness (R^2^ = 0.243, p< 0.069) with the increasing altitudinal gradient.

## Discussion

The community structure and regeneration status of the plant species could be predicted from the relative proportion of seedling, sapling, and adult stage individuals in the total populations of different species. We observed that the regeneration of the dominant species viz., *Betula utilis* and *Rhododendron campanulatum*, was higher, as compared to other associated species at both sites. *Betula utilis* exhibited a higher density in seedling and sapling stages in the upper temperate regions, located near settlement and highly disturbed area [76]. The population structure of the forest in this study reveals that the dominant species within the buffer zone of NDBR are distributed in all the life stage classes. However, the percentage density declines progressively from seedling to adult tree stages along the altitudinal gradient to the upper timberline. Among the plant species, *Betula utilis* had the highest tree density (724 & 368 trees ha^-1^), contributing to 44% and 36% of the total population at the TLR and BG sites, followed by *Pinus wallichiana* (24%) and *Cedrus deodara* (15%), respectively. The study also reveals that the density of shrubs and herb species was found to be higher in the BG site where the level of anthropogenic disturbance was lower than the TLR site of the buffer zone. Additionally, both the study areas showed a “good” number of seedling and sapling populations. Among the tree species, a higher density was represented by *Betula utilis* at both the sites, with the highest density (2,476 trees ha^-1^) recorded at TLR site. A lower density was recorded for *Vibernum* spp. (100 trees ha^-1^), followed by *Prunus cornuta* (50 trees ha^-1^). Some species, viz., *Populus ciliata*, *Picea smithiana* and *Pinus wallichiana*, were not found at the BG site but were present at the TLR site. While regeneration at both forested sites was found to be progressive, the TLR site had a higher density of seedlings and saplings. Human disturbances resulting from pilgrimage, nature tourism, livestock grazing, and the exploitation of local forest resources (i.e. tree cutting, lopping, debarking of trees, and extraction of other bio-resources, such as medicinal plants) may have affected the regeneration of species at the BG site.

This study showed huge variation in the density, basal area, and species diversity of a temperate forest ecosystem due to topography, altitudinal variation, forest type and micro climatic conditions (Table 9). The species richness reported in the present study is higher than what was reported in a nearby Kedarnath wildlife sanctuary (species = 75; tree = 3; shrub = 12; herb = 60) [76], which is located in the Western Himalayan Region of Uttarakhand, India, Additionally, the species richness is higher than the reported values for the temperate forests of Dudhatoli Garhwal Himalaya (species 268) [77], Azad Kashmir (species 200) [78], and the Naran Valley of Pakistan (species 198) [79]. However, the species richness value is lower than the moist temperate forest of Mandal-Chopta (species 300) in Garhwal Himalaya, Uttarakhand [80], and the temperate forest of NDBR (species 451) in the Western Himalayan Region of India [81]. Rosaceae (17.69%), Asteraceae (14.97%), Ranunculaceae (12.93%), Lamiaceae (7.48%), Poaceae (7.48%), Liliaceae (6.80%), and Polygonaceae (6.80%) were the most dominant species families in the temperate forests of the Western Himalayan Region. Similarly, Asteraceae and Lamiaceae have been reported as dominant families in the temperate forests of India and elsewhere [77, 79–82]. Shaheen et al (2011a) [83] also reported that Asteraceae (19%), Poaceae (13%), Ranunculaceae (11%), Rosaceae (8%), and Saxifragaceae (8%) were dominant families in the Western Himalayan Region of northern Pakistan. Dar et al (2012) [84] also reported that Asteraceae (260 species), Poaceae (160 species), Brassicaceae (115 species), Rosaceae (98 species), and Lamiaceae (88 species) were the major species families in Kashmir Himalaya. Hooker (1879) [85] stated that Orchidaceae, Fabaceae, Poaceae, Rubiaceae, Euphorbiaceae, Acanthaceae, Asteraceae, Cyperaceae, Lamiaceae, and Utricaceae are the diverse plant families of India. Therefore, the results of this study also support the concept that, across the various temperate forests of the world, a close similarity is evident at the family level. The highest shrub density was recorded for *Ribies alpester* and *Berberis jaeschkeana*, while the lowest was recorded for *Sorbaria tomentosa* and *Rubus niveus* in the BG and TLR sites, respectively. The dominant herbaceous species followed the sequence: *Fragaria nubicola*> *Oxalis corniculata* > *Geranium wallichianum*> *Anaphalis triplinervis*> *Impatiens sulcata* in the BG site. However, *Geranium himalayense* was recorded as the dominant species with *Oxalis corniculata* and *Fragaria nubicola* as co-dominant species at the TLR site.

**Table 9:** Comparisons of phytosociological attributes and species richness of different temperate forests.

Forest type	Region/locality	Altitude (m. asl.)	Total area sampled	Density (D)	Basal Area (BA)	Species richness	Source
Temperate forests	Kashmir Himalaya, India	1,550–3,250	111 (.25 ha)	103–1,201	19.4–51.9	177 = 14T+17S+146H	Dar et al. 2016
Temperate forests	Changbai Mountains, China	750–2,100	68 (0.04 ha)	-	-	213 = 37^T^+32^S^+144^H^	Bai et al. 2011
Temperate forest	Nanda Devi Biosphere Resrve, Chamoli district, Uttarakhand	2’350–3’900	30 (10X10 m)	599–1211	-	248 = 23^T^+44^S^+181^H^	Rawat et al. 2015
Sub-alpine region	Garhwal Himalaya, India	2,200–3,000	20 (50X50 m) 50 ha	-	-	90	Bisht and Bhat 2013
Temperate Deciduous forest	Denmark	-	50 ha	770	30.7	165	Borchsenius et al. 2004
Sub tropical to warm temperate	Central Himalaya	1,300–1,750	40 (0.01 ha)	540–1630	25–47.2	-	Chaturvedi & Singh 1987
Tropical semi evergreen	Manipur Northeast, India	-	20 (0.01 ha each)	10–675	-	123 = 17^T^+36^S^+70^H^	Devi and Yadava 2006
*Abies pindow*	Pithoragarh, Kumaun Himalaya	3100	3 (0.5 ha)	660	78.90	-	Dhar et al. 1997
Moist temperate forest	Mandal-Chopta, Garhwal Himalaya, Uttarakhand, India	1500–3000	NA	-	-	338	Gairola et al. 2010
Moist temperate forest	Western Himalaya Garhwal, Uttarakhand India	2400–2850	NA	380–1,180	41.25–86.56	65	Gairola et al. 2011a
Moist temperate forest	Western Himalaya, India	1500–2500	NA	990–1,470	35.08–84.25	125	Gairola et al. 2011b
Temperate forest	Mandal-CHopta, Garhwal Himalaya, Uttarakhand, India	1,500–2,850	NA	380–1,390	32.77–86.56	-	Gairola et al. 2012
Temperate forest	Northeast Spain	1500–2200	329	-	-	9	Gracia et al. 2007
Dry forest	Miombo, Zambia	1,292–1300	24 (0.25 ha)	308–736	5.6–27.5	83	Kalaba et al 2013
Temperate forest	Naran Valley, Pakistan	2,450–4100	144 (0.25 ha)	-	-	198 = 12^T^+20^S^+166^H^	Khan et al. 2011
Community temperate forest	Dolpha Mid-west, Nepal	1,900–2700	20 (0.01 ha)	2,090–2100	90.07–151.98	98	Kunwar & Sharma 2004
Temperate forest	Manang, Central Nepal	3,000–4,000	80 (0.01 ha)	-	-	168	Panthi et al.2007
Temperate forest	Arunachal Pradesh, India	350–700	60 (0.01 ha)	550–860	19.61–78.32	128 = 41^T^+22^S^+65^H^	Rana and Gairola 2009
Temperate forest	Garhwal Himalaya, India	500–6940	20 (0.01 ha)	1,090–1980	20.97–40.19	8–19	Raturi 2012
Wet temperate forest	Abottabad, Pakistan	800–2500	NA	-	-	167	Saima et al. 2010
Temperate forest	Kumaun Himalaya	1280–2227	48 (0.01 ha)	-	-	7–21	Saxena & Singh 1984
Temperate forest	Central Himalaya, India	1400–2700	60 (0.01 ha)	20–170	-	116 = 16^T^+35^S^+65^H^	Semwal et al.2010
Temperate forest	Western Himalaya, northen Pakistan	>3300	30 (0.01 ha)	-	-	83	Shaheen et al. 2011a
Temperate alpine pasture	Western Himalaya, Pakistan	2600–3500	20.5 ha	-	-	69	Shaheen et al. 2011b
Moist temperate forest	Western Himalaya, Kashmir	1700–2600	180 (900 m2 each)	90–227	42.32–105.29	122	Shaheen et al. 2012
Moist temperate forest	Dudhatoli Garhwal Himalaya	1800–3000	NA	-	-	268	Sharma et al. 2013
Alpine zone	Northwest Yunnan, China	3800–5200	70 (0.036)	-	-	369	Sherman et al. 2012
Temperate forest	Shimla, Himachal Pradesh, India	1650–2295	36 (0.01 ha)	4,217–7765	18.49–52.54	55 = 6^T^+14^S^+35^H^	Singh & Gupta 2009
Temperate: Evergreen deciduous & coniferous	Mt.Emei, Sichuen, China	680–3099	10 (0.02–0.04)	-	-	122	Tang & Ohsawa 1997
Temperate forest	Azad Kashmir, Pakistan	-	70 (0.01 ha)	-	-	200	Tanvir et al. 2014
Temperate forest	Baihua Mountain, China	750–2043	61 (0.02 ha)	-	-	71	Zhang et al. 2013

Soil characteristics may severely influence the vegetation, while vegetation structure and composition also affect the soil properties. The selective absorption of nutrients by different tree species and their capacity to return nutrients to the soil bring about changes in soil properties [8]. The soil texture is an important factor which indicates how well a particular soil type can hold water. Values regarding water holding capacity varied from 28.70±3.35% to 33.50±4.54% and 40.58± 6.92% to 49.94±5.63% at the TLR and BG sites, respectively. The values for water holding capacity in the present study are within the line of values that were reported earlier by various workers from the temperate region of Garhwal Himalaya [5, 86]. Within the soil, the overall water holding capacity increased with the increasing clay content and decreased with the decreasing sand content. The calculated values for moisture content ranged between 46.49±3.85% to 63.77 ± 4.77% and were slightly higher than the values that were reported by Khera et al. (2001) [87] for Kumaun Himalaya and Nazir (2009) [86] for Garhwal Himalaya. Clay, silt, and sand contents in the soil of the studied sites ranged from 0.87±0.12% to 0.88±0.06%, 34.65±2.71% to 50.4±5.45%, and 46.88±2.70% to 63.98±8.27% for the BG site. Comparatively, the TLR site exhibited a range of 6.88±2.80% to 7.82±1.41%, 27.20±3.16% to 33.01±4.11%, and 60.11±4.13% to 64.99±3.95%, respectively. The soil pH in the BG and TLR sites ranged between 6.28±0.22 and 6.94±0.15, indicating that the soil was slightly acidic to near the neutral value, which was assumed to be the most favorable range for nutrient availability within the stand. In general, pH values for the northern aspect were higher compared to the contrasting southern aspect, which could be attributed to the presence of coniferous leaf litter, stone rocks, and more precipitation. The soil phosphorus content among all the stands was found in decreasing order with the soil depth. The phosphorus content ranged between 0.09±0.02% to 0.11±0.01% at both sites. Values for available K were observed between 1.23±0.69% and 1.33±0.27% at the BG site, while the TLR site showed a range between 1.32±0.56% and 1.47±0.72%. The soil nitrogen concentration ranged between 0.170±0.07% and 0.218±0.06% at both sites. The values of total N in the present study are higher than the values recorded by Khera et al. (2001) [87], Srivastava et al. (2005) [88], Semwal et al. (2010) [89], Pandey et al. (2001) [90], Sharma et al. (2010) [5], Thadani and Ashton (1995) [91] and Nazir (2009) [86]. This could be attributed to a higher water holding capacity and an increase in the accumulation of litter and humus in the upper layers of forest soil. The present results revealed that soil carbon (0.89±0.11% to 1.06±0.25%) and organic matter (1.54±0.20% to 1.82±0.44%) has increased with an increasing altitude. Similar findings were reported by various workers in earlier studies on mountain terrain [92–104]. Hence, increasing trends of soil organic matter (%) with increasing altitudes may be due to the constant carbon inputs and decreasing rate of carbon loss at different altitudes (Table 6). The study revealed that the intense recruitment of seedling and sapling stage *Betula utilis* and *Rhododendron campanulatum* individuals along the altitudinal gradient to the upper timberline may be due to the more favorable climatic conditions that have been provided for growth during past decades, and/or to land use changes in the high-altitude regions that have dramatically increased the potential for future vegetation advancement. This study also exhibited a continuous decrease in the diameter (CBH) of trees and augmentation of sapling and seedlings of *Betula utilis* and *Rhododendron campanulatum* along an altitudinal gradient. This is a clear indication that these species may gradually move upward to the higher altitude. With increased global warming, the vegetation of high-altitude areas would be expected to advance upwards, but the changes observed in both study areas have been limited to enhance the recruitment and growth of species rather than vegetation advancement. However, to confirm the shifting of vegetation in NDBR, the long-term studies are required through establishment of permanent plots with manual, as well as automatic, weather data recording set-ups. The upper limit of survival of the plant species is still unknown for these areas due to a lack of long-term scientific data is and the fact that the region has been under high anthropogenic pressure in the recent past.

## Conclusion

The study area is comprised of a variety of ecologically important plant species, many of which are listed under different threatened categories of IUCN. However, the area also has significance for religious pilgrims (BG site, enroute of the Sikh shrine), research expeditions (TLR site, enroute of Nanda Devi National Park), and providing opportunities to rural communities who depend on it for its forest bioresources (fuelwood, fodder, timber, NTFPs, MAPs etc.). Our study observed the progressive growth of seedling and sapling individuals of dominant species in the temperate zone due to changes in the micro-climatic condition of the region. Therefore, the regular, long-term monitoring of plant communities at different altitudes is urgently needed to project the change in forest structure, composition, and regeneration patterns in the temperate region of the western Himalayas. Thus, sustainable management strategies and appropriate policy interventions could be designed to highlight the biodiversity richness, anthropogenic pressure, and bioresource utilization, to increase ecological awareness and share the benefits of a sustainable livelihood on a regional and/or global scale.

## Supporting information

S1 Table Diversity and distribution of vascular plants in BG and TLR, sites of Nanda Devi Biosphere Reserve, Western Himalaya. (DOCX)

S2 Table Distribution of families in BG and TLR sites, Nanda Devi Biosphere Reserve, Western Himalaya. (DOCX)
