# Supplementary material for: Vegetation dynamics and soil nutrient availability in a temperate forest along altitudinal gradient of Nanda Devi Biosphere Reserve, Western Himalaya, India
Source: PLoS One. 2022 Oct 7;17(10):e0275051. doi: 10.1371/journal.pone.0275051 (PMC9544032; doi:10.1371/journal.pone.0275051)
Supplement: S1 Table — (DOCX) [file pone.0275051.s001.docx]

**Supplementary material**

**Table S1.** Diversity and distribution of vascular plants in BG and TLR, sites of Nanda Devi Biosphere Reserve, Western Himalaya.

| **Family** | **Genera** | **Species** | **Life forms** | **Altitudinal range** | **Sites** |
| --- | --- | --- | --- | --- | --- |
| **Angiosperms** |  |  |  |  |  |
| **Ranunculaceae** | **Aconitum** | *Aconitum atrox* (Bruhl) Mukerji | H | 3000-3800 | BG, TLR |
|  | **Actaea** | *Actaea spicata* (L.) Royle var. | H | 2500-3400 | TLR |
|  | **Anemone** | *Anemone obtusiloba* D.Don | H | 3200-4400 | BG, TLR |
|  |  | *Anemone tetrasepala* Royle | H | 3000-4400 | TLR |
|  | **Clematis** | *Clematis acumminata* DC. | CL | 2500-3600 | BG, TLR |
|  |  | *Clematis montana* Buch.-Ham. ex DC. | CL | 2800-3600 | TLR |
|  | **Delphinium** | *Delphinium vestitum* Wall. ex Royle | H | 3500-4000 | BG |
|  | **Ranunculus** | *Ranunculus diffusus* DC. | H | 2800-3400 | BG, TLR |
|  |  | *Ranunculus hirtellus* Royle | H | 2500-3800 | TLR |
|  | **Thalictrum** | *Thalictrum alpinum* L. | H | 3200-3800 | BG, TLR |
|  |  | *Thalictrum minus* L. | H | 3000-3600 | TLR |
| **Berberidaceae** | **Berberis** | *Berberis aristata* DC. | SH | 2400-3000 | BG, TLR |
|  |  | *Berberis chitria* Buch.-Ham. ex Lindl. | SH | 2500-3200 | BG, TLR |
|  |  | *Berberis jaeschkeana* C.K.Schneid. | SH | 3000-3600 | TLR |
| **Podophyllaceae** | **Podophyllum** | *Podophyllum hexandrum* Royle | H | 3000-3600 | BG, TLR |
| **Papaveraceae** | **Meconopsis** | *Meconopsis aculeata* Royle | H | 3000-3800 | BG, TLR |
|  | **Corydalis** | *Corydalis falconeri* Hook. f. & Thomson | H | 3000-3600 | BG, TLR |
| **Brassicaceae** | **Cardamine** | *Cardamine impatiens* L. | H | 2500-3600 | TLR |
|  | **Arabidopsis** | *Arabidopsis thaliana* (L.) Heynh. | H | 3000-3600 | TLR |
|  | **Erysimum** | *Erysimum repandrum* L. | H | 3400-4000 | BG, TLR |
|  | **Capsella** | *Capsella bursa-pastoris* (L.) Medik. | H | 2700-4000 | BG, TLR |
|  | **Megacarpaea** | *Megacarpaea* *polyandra* Benth. ex Madden | H | 3000-3500 | BG |
|  | **Thlaspi** | *Thlaspi cochleariforme* DC. | H | 3200-3800 | TLR |
| **Violaceae** | **Viola** | *Viola biflora* L. | H | 2500-4000 | BG, TLR |
| **Caryophyllaceae** | **Arenaria** | *Arenaria ciliolata* Edgew. & Hk.f | H | 3000-4000 | BG |
|  | **Silene** | *Silene edgewarthi* Bocquet | H | 2800-3300 | BG, TLR |
|  |  | *Silene vulgaris* (Moench) Garcke | H | 3000-3600 | BG, TLR |
|  | **Stellaria** | *Stellaria himalayensis* Majumdar | H | 3400-4000 | BG |
|  | **Cerastium** | *Cerastium cerastioides* (L.) | H | 2400-3800 | TLR |
| **Hypericaceae** | **Hypericum** | *Hypericum japonicum* Thunb. | H | 2400-3600 | TLR |
| **Malvaceae** | **Malva** | *Malva rotundifolia* L. | H | 3000-3600 | TLR |
| **Geraniaceae** | **Geranium** | *Geranium nepalense* Sweet | H | 3000-3500 | BG, TLR |
|  |  | *Geranium wallichianum* D.Don ex Sweet | H | 2800-3800 | BG, TLR |
|  | **Erodium** | *Erodium stephanianum* Willd. | H | 2400-2800 | TLR |
| **Oxalidaceae** | **Oxalis** | *Oxalis acetosella* L. | H | 2800-3600 | BG, TLR |
|  |  | *Oxalis corniculata* L. | H | 2500-3800 | BG, TLR |
| **Balsaminaceae** | **Impatiens** | *Impatiens thomsonii* Hook.f. | H | 3000-4000 | BG, TLR |
|  |  | *Impatiens sulcata* Wall. | H | 3200-4000 | BG, TLR |
| **Rutaceae** | **Skimmia** | *Skimmia anquitilia* N.P. Tayler & Airy Shaw | SH | 3000-3600 | BG, TLR |
|  | **Boeninghausenia** | *Boeninghausenia albiflora* (Hk.) | H | 2400-2800 | TLR |
| **Aceraceae** | **Acer** | *Acer acuminatum* Wall. ex D.Don | T | 2600-3500 | BG, TLR |
|  |  | *Acer caesium* Wall. ex Brandis | T | 2500-3400 | BG, TLR |
| **Aquifoliaceae** | **Ilex** | *Ilex dipyrena* Wall. | T | 2800-3400 | BG |
| **Fabaceae** | **Astragalus** | *Astragalus chlorostachys* Lindl. | H | 3200-3800 | BG, TLR |
|  |  | *Astragalus himalayanus* Klotzsch | H | 2600-4000 | BG, TLR |
|  | **Desmodium** | *Desmodium elegans* DC. | SH | 2000-3000 | BG |
|  | **Piptanthus** | *Piptanthus nepalensis* (Hook) D.Don | SH | 2600-3400 | TLR |
|  | **Trifolium** | *Trifolium repens* L. | H | 3000-3600 | TLR |
|  | **Caragana** | *Caragana nubigena* Bunge | SH | 3200-4500 | TLR |
|  | **Trigonella** | *Trigonella emodi* Benth. | H | 2500-3000 | TLR |
|  | **Lotus** | *Lotus corniculatus* L. | H | 3400-4000 | BG, TLR |
|  | **Indigofera** | *Indigofera hebepetala* Benth. Ex Baker | SH | 2500-3000 | BG, TLR |
| **Rosaceae** | **Cotoneaster** | *Cotoneaster acuminatus* Lindl. | SH | 3000-3800 | BG, TLR |
|  |  | *Cotoneaster microphyllus* Wall. ex Lindl. | SH | 2500-3800 | BG, TLR |
|  | **Fragaria** | *Fragaria daltoniana* J. Gay | H | 2500-3500 | TLR |
|  |  | *Fragaria nubicola* (Hook. f.) Lindl. ex Lacaita | H | 2800-3800 | BG, TLR |
|  | **Geum** | *Geum elatum* Wall ex G.Don | H | 2700-4000 | BG, TLR |
|  | **Prunus** | Prunus *cornuta* (Wall ex Royle) Steud. | T | 2400-3200 | BG |
|  | **Potentilla** | *Potentilla atrosanguinea* Lodd. ex Lehm. | H | 3000-4200 | BG, TLR |
|  |  | *Potentilla microphylla* D.Don | H | 3300-4500 | BG, TLR |
|  | **Rosa** | *Rosa macrophylla* Lindl. | SH | 2400-3800 | BG, TLR |
|  |  | *Rosa webbiana* Wall ex Royle | SH | 2100-3600 | BG, TLR |
|  | **Rubus** | *Rubus nepalensis* (Hook. f.) Kuntze | H | 2800-3200 | BG, TLR |
|  |  | *Rubus niveus* Thunb. | SH | 2500-3600 | BG, TLR |
|  | **Sibbaldia** | *Sibbaldia cuneata* Hornem. ex Kuntze | H | 3300-3800 | TLR |
|  | **Sorbus** | *Sorbus cuspidata* (Spach) Hedl. | T | 2600-3600 | BG, TLR |
|  | **Pyrus** | *Pyrus pashia* Buch.-Ham.ex D. Don | T | 2400-2700 | TLR |
|  | **Agrimonia** | *Agrimonia pilosa* Ledeb | H | 2400-2800 | BG, TLR |
|  | **Filipendula** | *Filipendula vestita* (Wall. Ex G. Don) Maxim. | H | 3000-4000 | BG, TLR |
|  | **Prinsepia** | *Prinsepia utilis* Royle | SH | 2200-2800 | BG, TLR |
|  | **Spiraea** | *Spiraea bella* Sims. | SH | 3000-3600 | BG, TLR |
| **Saxifragaceae** | **Bergenia** | *Bergenia stracheyi* (Hook.f. & Thomson) Engl. | H | 3000-4500 | BG, TLR |
|  |  | *Bergenia ligulata* (Wall.) Engl. | H | 2800-3200 | BG, TLR |
|  | **Saxifraga** | *Saxifraga brunonis* Wall. ex Ser. | H | 2800-3600 | TLR |
|  |  | *Saxifraga diversifolia* Wall. ex Ser. | H | 3000-3800 | BG, TLR |
| **Grossulariaceae** | **Ribes** | *Ribes* *orientale* Desf. | SH | 2600-3200 | BG, TLR |
|  |  | *Ribes alpestre* | SH | 2600-3000 | BG, TLR |
| **Crassulaceae** | **Rhodiola** | *Rhodiola sinuata* (Royle ex Edgew.) S.H. Fu | H | 3000-3800 | BG, TLR |
|  | **Sedum** | *Sedum quadrifidum* Pall. | H | 3000-4000 | BG, TLR |
|  | **Rosularia** | *Rosularia rosulata* (Edgew.) Obha. | H | 2800-3300 | BG, TLR |
| **Onagraceae** | **Circaea** | *Circaea alpina* L. | H | 2500-3500 | TLR |
|  | **Epilobium** | *Epilobium latifolium* L. | H | 3200-4200 | TLR |
|  | **Oenothera** | *Oenothera rosea* Ait. | H | 2000-2800 | TLR |
| **Apiaceae** | **Acronema** | *Acronema hookeri* (C.B.Clarke) H.Wolff | H | 3000-4000 | BG, TLR |
|  | **Angelica** | *Angelica archangelica* L. | H | 3000-3800 | BG, TLR |
|  |  | *Angelica glauca* Edgew. | H | 3000-4000 | BG, TLR |
|  | **Bupleurum** | *Bupleurum hamiltonii* N.P.Balakr. | H | 3000-3800 | TLR |
|  |  | *Bupleurum longicaule* Wall. ex DC. | H | 3200-3800 | TLR |
|  | **Chaerophyllum** | *Chaerophyllum reflexum* Lindl. | H | 2300-3000 | TLR |
|  | **Carum** | *Carum carvi* L. | H | 2500-3800 | BG, TLR |
|  | **Heracleum** | *Heracleum canescens* Lindl. | H | 3000-4200 | BG, TLR |
|  | **Pleurospermum** | *Pleurospermum brunonis* (DC.) C.B.Clarke | H | 3000-4000 | TLR |
|  |  | *Pleurospermum angelicoides* (Wall. ex DC.) Benth. ex C.B. Clarke | H | 2800-4000 | BG, TLR |
|  | **Selinum** | *Selinum candollei* DC. | H | 3000-4000 | GB, TLR |
|  |  | *Selinum wallichianum* (DC.) Raizada & H.O. Saxena | H | 3000-3800 | BG |
|  | **Pimpinella** | *Pimpinella acuminata* (Edgew.) Cl. | H | 2500-3000 | TLR |
| **Caprifoliaceae** | **Lonicera** | *Lonicera myrtillus* Hook f. & Thomson | SH | 3000-3800 | BG, TLR |
|  | **Viburnum** | *Viburnum grandiflorum* Wall. ex DC. | SH | 3000-3500 | BG, TLR |
| **Rubiaceae** | **Galium** | *Galium aparine* L. | H | 3000-4000 | TLR |
|  | **Leptodermis** | *Leptodermis lanceolata* Wall. | SH | 3000-4000 | TLR |
|  | **Rubia** | *Rubia cordifolia* L. | H | 3200-3800 | BG, TLR |
| **Valerianaceae** | **Nardostachys** | *Nardostachys jatamansi* (D. Don) DC. | H | 3300-4000 | BG, TLR |
|  | **Valeriana** | *Valeriana hardwickii* Wall. | H | 3000-4200 | BG, TLR |
| **Morinaceae** | **Morina** | *Morina longifolia* Wall. ex DC. | H | 3000-3800 | BG, TLR |
| **Asteraceae** | **Anaphalis** | *Anaphalis contorta* (D.Don) Hook.f | H | 3000-4000 | BG, TLR |
|  |  | *Anaphalis royleana* DC. | H | 2800-3800 | BG, TLR |
|  | **Artemisis** | *Artemisia nilagirica* (Cl.) Pamp. | H | 2300-3300 | TLR |
|  | **Erigeron** | *Erigeron alpinus* L. | H | 3000-3800 | TLR |
|  | **Gerbera** | *Gerbera kunzeana* A.Braun & Aschers | H | 3000-4000 | TLR |
|  | **Ligularia** | *Ligularia amplexicaulis* DC. | H | 3000-3800 | BG, TLR |
|  | **Myractis** | *Myractis nepalensis* Less. | H | 3200-4000 | TLR |
|  | **Saussurea** | *Saussurea auriculata* (DC.) Sch.Bip. | H | 2800-3600 | BG, TLR |
|  |  | *Saussurea nepalensis* Spreng. | H | 3000-3600 | BG, TLR |
|  | **Tagetes** | *Tagetes minuta* L. | H | 2200-2800 | TLR |
|  | **Senecio** | *Senecio candolleanus* Wall. | H | 3000-3800 | BG, TLR |
|  |  | *Senecio graciliflorus* (Wall.) DC. | H | 3000-3800 | BG, TLR |
|  | **Conyza** | *Conyza stricta* Willd. | H | 2200-2800 | TLR |
|  | **Taraxacum** | *Taraxacum officinale* Webb | H | 2800-3800 | BG, TLR |
| **Campanulaceae** | **Campanula** | *Campanula argyrotricha* Wall.ex DC. | H | 3000-4000 | TLR |
|  | **Cyananthus** | *Cyananthus microphyllus* Edgew. | H | 3200-4000 | BG, TLR |
|  |  | *Cyanathus integer* Wall.ex Benth. | H | 2500-3500 | TLR |
| **Ericaceae** | **Gaultheria** | *Gaultheria nummularioides* D.Don | H | 3000-3600 | BG, TLR |
|  |  | *Gaultheria trichophylla* Royle | H | 3000-3800 | TLR |
|  | **Rhododendron** | *Rhododendron anthopogon* D.Don | SH | 3000-4500 | BG, TLR |
|  |  | *Rhododendron campanulatum* D.Don | T | 3000-4000 | BG, TLR |
| **Primulaceae** | **Androsace** | *Androsace sarmentosa* Wall. | H | 3000-4000 | TLR |
|  | **Primula** | *Primula denticulata* Sm. | H | 2500-3600 | BG, TLR |
|  |  | *Primula edgeworthii* Pax | H | 3200-3800 | BG, TLR |
| **Gentianaceae** | **Gentiana** | *Gentiana argentea* Royle ex D.Don | H | 3000-4000 | BG, TLR |
|  |  | *Gentiana carinata* Griseb. | H | 3000-4000 | TLR |
|  | **Swertia** | *Swertia alata* Royle ex D.Don | H | 2800-3600 | TLR |
| **Scrophulariaceae** | **Falconeria** | *Falconeria himalaica* HooK.f. | H | 3200-3800 | TLR |
|  | **Hemiphragma** | *Hemiphragma heterophyllum* Wall. | H | 3000-4000 | TLR |
|  | **Pedicularis** | *Pedicularis gracilis* Wall. ex Benth. | H | 3300-4200 | TLR |
|  | **Picrorhiza** | *Picrorhiza kurrooa* Royle ex Benth. | H | 3000-4200 | BG, TLR |
|  | **Scrophularia** | *Scrophularia himalensis* Royle ex Benth. | H | 2600-3400 | BG, TLR |
|  | **Mazus** | *Mazus pumilus* (Burm.f.) Steen | H | 2200-2800 | TLR |
| **Lamiaceae** | **Ajuga** | *Ajuga brachystemon* Maxim. | H | 2400-3500 | TLR |
|  | **Clinopodium** | *Clinopodium umbrosum* (M.Bieb.) Kuntze | H | 3000-4000 | TLR |
|  | **Lamimum** | *Lamium album* L. | H | 2800-3600 | TLR |
|  | **Leonurus** | *Leonurus cardiaca* L. | H | 3000-4000 | BG |
|  | **Nepeta** | *Nepeta govaniana* (Wall. ex Benth.) Benth. | H | 2800-3600 | BG, TLR |
|  | **Phlomis** | *Phlomis bracteosa* Royle ex Benth. | H | 2500-3500 | BG, TLR |
|  | **Salvia** | *Salvia nubicola* Wall. ex Sweet | H | 3000-3800 | BG, TLR |
| **Polygonaceae** | **Aconogonon** | *Aconogonon rumicifolium* (Royle ex Bab.) H.Hara | H | 3000-3800 | BG, TLR |
|  | **Bistorta** | *Bistorta affinis* (D.Don) Greene | H | 3200-4200 | BG, TLR |
|  |  | *Bistorta emodi* (Meisn.) H.Hara | H | 3000-4200 | BG, TLR |
|  | **Oxyria** | *Oxyria digyna* (L.) Hill | H | 2500-4000 | BG, TLR |
|  | **Persicaria** | *Persicaria alpina* (All.) H.Gross | H | 3000-4000 | BG |
|  | **Polygonum** | *Polygonum delicatulum* Meisn. | H | 3000-4000 | BG, TLR |
|  |  | *Polygonum polystachyum* Wall ex Meissner | H | 3000-4500 | BG, TLR |
|  | **Rheum** | *Rheum australe* D. Don | H | 3000-4000 | BG, TLR |
|  |  | *Rheum webbianum* Royle | H | 3000-3800 | BG, TLR |
|  | **Rumex** | *Rumex nepalensis* Spreng. | H | 2000-4000 | BG, TLR |
| **Eleagnaceae** | **Hippophae** | *Hippophae salicifolia* D.Don | SH | 2500-3200 | BG, TLR |
|  |  | *Euphorbia stracheyi* Boiss. | H | 3200-4200 | TLR |
| **Urticaceae** | **Urtica** | *Urtica dioica* L. | H | 2500-3600 | BG, TLR |
|  |  | *Urtica parviflora* Roxb. | H | 2000-3600 | TLR |
| **Betulaceae** | **Betula** | *Betula utilis* D.Don | T | 3000-4200 | BG, TLR |
| **Salicaceae** | **Salix** | *Salix denticulata* Andersson | SH | 2500-3400 | BG, TLR |
|  |  | *Salix elegans* Wall. ex Anderss. | H | 2400-3200 | TLR |
|  |  | *Salix sikkimensis* Anders. | T | 3200-3700 | BG |
|  | **Populus** | *Populus ciliata* Wall. ex Royle | T | 2400-3400 | TLR |
| **Orchidaceae** | **Cypripedium** | *Cypripedium elegans* Reichb.f. | H | 2800-3500 | BG |
|  |  | *Cypripedium himalaicum* Rolfe | H | 3000-3500 | BG |
|  | **Habenaria** | *Habenaria edgeworthii* Hook.f.ex Collet | H | 2500-3300 | BG |
|  | **Dactylorhiza** | *Dactylorhiza hatagirea* (D.Don) Soó | H | 3200-4000 | BG, TLR |
|  | **Goodyera** | *Goodyera fusca* (Lindl.) Hook.f. | H | 3000-3800 | TLR |
|  | **Malaxis** | *Malaxis muscifera* (Lindl.) Kutze. | H | 2500-3600 | BG |
|  | **Neottia** | *Neottia listeroides* Lindl. | H | 2600-3300 | TLR |
|  | **Peristylus** | *Peristylus duthiei* (Hook.f.) Deva & H.B.Naithani | H | 3000-3600 | TLR |
| **Zingiberaceae** | **Roscoea** | *Roscoea alpina* Royle | H | 3000-3600 | BG, TLR |
| **Amaryllidaceae** | **Allium** | *Allium stracheyi* Baker | H | 3000-3600 | BG, TLR |
|  |  | *Allium wallichii* Kunth | H | 2500-4000 | BG |
| **Liliaceae** | **Asparagus** | *Asparagus filicinus* Buch-Ham. ex D.Don | SH | 2500-3300 | BG |
|  | **Clintonia** | *Clintonia udensis* Trautv. & Mey. var. alpina (Kunth ex Baker) Hara | H | 3000-3500 | BG, TLR |
|  | **Fritillaria** | *Fritillaria roylei* Hook. | H | 3200-4000 | BG |
|  | **Polygonatum** | *Polygonatum verticillatum* (L.) All. | H | 2200-4000 | BG |
|  |  | *Polygonatum cirrhifolium* (Wall) Royle | H | 2800-3800 | BG |
|  | **Smilacina** | *Smilacina purpurea* Wall. | H | 2600-3000 | BG, TLR |
|  | **Trillidium** | *Trillidium govanianum* Wall. ex D.Don | H | 2500-3600 | BG, TLR |
| **Juncaceae** | **Juncus** | *Juncus concinnus* D.Don | H | 3000-4000 | BG, TLR |
|  |  | *Juncus himalensis* Klotzsch | H | 3000-4200 | TLR |
| **Areceae** | **Arisaema** | *Arisaema intermedium* Blume | H | 2800-3800 | BG, TLR |
|  |  | *Arisaema jacquemontii* Blume | H | 2500-3400 | BG, TLR |
| **Cyperacaeae** | **Carex** | *Carex duthiei* Cl. | SH | 3400-3800 | BG |
|  |  | *Carex inanis* Kunth. | SH | 2800-3600 | TLR |
| **Poaceae** | **Agrostis** | *Agrostis canina* L. | G | 3200-4000 | BG, TLR |
|  |  | *Agrostis pilosula* Trin. | G | 3200-4200 | BG, TLR |
|  | **Andropgon** | *Andropgon munroi* Cl. | G | 3000-3500 | TLR |
|  | **Calamagrostis** | *Calamagrostis lahulensis* G.Singh | G | 3200-4000 | TLR |
|  | **Festuca** | *Festuca gigantea* (L.) Vill. | G | 3000-3800 | TLR |
|  |  | *Festuca kashmiriana* Stapf | G | 3200-3600 | TLR |
|  | **Poa** | *Poa alpina* L. | G | 3000-3800 | BG, TLR |
|  |  | *Poa annua* L. | G | 3000-3600 | BG, TLR |
|  | **Thamnocalamus** | *Thamnocalamus spathiflorus* (Trin.) Munro | SH | 3000-4000 | BG, TLR |
|  | **Trisetum** | *Trisetum clarkei* (Hook.f.) R.R.Stewart | G | 3000-4000 | TLR |
| **Gymnosperms** |  |  |  |  |  |
| **Ephedraceae** | **Ephedra** | *Ephedra gerardiana* Wall. Ex Stapf. | SH | 3300-4200 | TLR |
| **Pinaceae** | **Abies** | *Abies pindrow* (Royle ex D.Don) Royle | T | 3000-3800 | BG, TLR |
|  | **Picea** | *Picea smithiana* (Wall.) Boiss. | T | 3200-3800 | BG, TLR |
|  | **Pinus** | *Pinus wallichiana* A.B. Jacks. | T | 3000-3500 | BG, TLR |
|  | **Cedrus** | *Cedrus deodara* (Roxb.) G.Don | T | 3000-3800 | BG, TLR |
| **Cupressaceae** | **Juniperus** | *Juniperus communis* L. Ver Saxatilis Pallas | SH | 3000-4000 | BG, TLR |
|  |  | *Juniperus indica* Bertol. | SH | 3200-3800 | BG, TLR |
|  | **Cupressus** | *Cupressus torulosa* D.Don | T | 2500-3000 | BG, TLR |
| **Taxaceae** | **Taxus** | *Taxus baccata* L. | T | 3000-3600 | BG, TLR |
| **Pteridophytes** |  |  |  |  |  |
| **Osmundaceae** | **Osmunda** | *Osmunda claytoniana* L. | PT | 3000-3800 | BG, TLR |
| **Sinopteridaceae** | **Notholaena** | *Notholaena morantae* (L.) Desv. | PT | 2800-3800 | TLR |
|  | **Cheilathes** | *Cheilathes albomarginata* Cl. | PT | 2400-3000 | TLR |
| **Aspleniaceae** | **Aspleninum** | *Aspleninum dalhousiae* Hk. | PT | 2400-3300 | TLR |
|  |  | *Aspleninum trichomones* L. | PT | 2800-3600 | BG, TLR |
| **Athyriaceae** | **Athyrium** | *Athyrium foliolosum* Wall. ex Sim. | PT | 2400-3300 | BG, TLR |
|  |  | *Athyrium schimperi* Moung. ex Fee | PT | 2500-3400 | TLR |
|  | **Diplazium** | *Diplazium esculentum* (Retz.) Sw | PT | 2200-2800 | TLR |
|  | **Gymnocarpium** | *Gymnocarpium dryopteris* (L.) | PT | 3000-3500 | BG, TLR |
|  | **Cystopteris** | *Cystopteris fragilis* (L.) Bernh. | PT | 3200-4000 | TLR |
| **Dryopteridaceae** | **Dryopteris** | *Dryopteris crysocoma* (Christ) C. Chr. | PT | 2500-3300 | BG, TLR |
|  | **Polystichum** | *Polystichum squarrosum* (D. Don) Fee | PT | 2200-3000 | TLR |
| **Davalliaceae** | **Araiostegia** | *Araiostegia beddomei* (Hope) Ching | PT | 2300-2800 | BG, TLR |
|  | **Leucostegia** | *Leucostegia immersa* Presl. | PT | 2500-2800 | TLR |
| **Polypodiaceae** | **Drynaria** | *Drynaria mollis* Bedd. | PT | 2350-4000 | TLR |
|  | **Lepisorus** | *Lepisorus jakonensis* (Blanf.) Ching | PT | 2400-2800 | TLR |
|  | **Phymatopteris** | *Phymatopteris ebenipes* (Hk.) Pichi Sermoli | PT | 3000-3800 | TLR |

Abbreviations used: T= Tree, SH= Shrubs; H= Herbs, CL= Climber; G= Grass; PT= Pteridophytes, BG= Bhyundar-Ghangaria site; TLR= Tolma-Lata-Raini site
