# Supplementary material for: Vegetation dynamics and soil nutrient availability in a temperate forest along altitudinal gradient of Nanda Devi Biosphere Reserve, Western Himalaya, India
Source: PLoS One. 2022 Oct 7;17(10):e0275051. doi: 10.1371/journal.pone.0275051 (PMC9544032; doi:10.1371/journal.pone.0275051)
Supplement: S2 Table — (DOCX) [file pone.0275051.s002.docx]

**Supplementary material**

**Table S2. Distribution of families in BG and TLR sites, Nanda Devi Biosphere Reserve, Western Himalaya.**

| **Families** | **No. of genera** | | **No. of species** | | **Species %** | | **Total species occurrence** |
| --- | --- | --- | --- | --- | --- | --- | --- |
|  | **BG site** | **TLR Site** | **BG site** | **TLR Site** | **BG site** | **TLR Site** |  |
| **Angiosperms** | | | | | | | |
| *Ranunculaceae* | 6 | 3 | 9 | 10 | 6.12 | 6.80 | 12.93 |
| *Berberidaceae* | 1 | 1 | 3 | 2 | 2.04 | 1.36 | 3.40 |
| *Podophyllaceae* | 1 | 1 | 1 | 1 | 0.68 | 0.68 | 1.36 |
| *Papaveraceae* | 1 | 1 | 1 | 1 | 0.68 | 0.68 | 1.36 |
| *Brassicaceae* | 1 | 1 | 1 | 2 | 0.68 | 1.36 | 2.04 |
| *Violaceae* | 1 | 1 | 1 | 1 | 0.68 | 0.68 | 1.36 |
| *Polyglaceae* | 1 | 1 | 1 | 1 | 0.68 | 0.68 | 1.36 |
| *Caryophyllaceae* | 1 | 2 | 1 | 4 | 0.68 | 2.72 | 3.40 |
| *Geraniaceae* | 1 | 0 | 3 | 0 | 2.04 | 0.00 | 2.04 |
| *Balsaminaceae* | 1 | 0 | 2 | 0 | 1.36 | 0.00 | 1.36 |
| *Aceracear* | 1 | 1 | 2 | 1 | 1.36 | 0.68 | 2.04 |
| *Fabaceae* | 2 | 3 | 5 | 3 | 3.40 | 2.04 | 5.44 |
| *Rosaceae* | 5 | 6 | 16 | 10 | 10.88 | 6.80 | 17.69 |
| *Saxifragaceae* | 2 | 1 | 4 | 2 | 2.72 | 1.36 | 4.08 |
| *Grossulariaceae* | 0 | 1 | 0 | 4 | 0.00 | 2.72 | 2.72 |
| *Crassulaceae* | 0 | 2 | 0 | 6 | 0.00 | 4.08 | 4.08 |
| *Onagraceae* | 1 | 1 | 2 | 1 | 1.36 | 0.68 | 2.04 |
| *Apiaceae* | 3 | 3 | 5 | 3 | 3.40 | 2.04 | 5.44 |
| *Caprifoliaceae* | 0 | 2 | 0 | 2 | 0.00 | 1.36 | 1.36 |
| *Rubiaceae* | 1 | 0 | 2 | 0 | 1.36 | 0.00 | 1.36 |
| *Valerianaceae* | 1 | 1 | 1 | 1 | 0.68 | 0.68 | 1.36 |
| *Dipsacaceae* | 0 | 1 | 0 | 2 | 0.00 | 1.36 | 1.36 |
| *Asteraceae* | 8 | 6 | 13 | 9 | 8.84 | 6.12 | 14.97 |
| *Campanulaceae* | 1 | 1 | 2 | 2 | 1.36 | 1.36 | 2.72 |
| *Ericaceae* | 2 | 1 | 3 | 2 | 2.04 | 1.36 | 3.40 |
| *Primulacaeae* | 1 | 1 | 3 | 3 | 2.04 | 2.04 | 4.08 |
| *Gentianaceae* | 3 | 2 | 5 | 3 | 3.40 | 2.04 | 5.44 |
| *Boraginaceae* | 2 | 2 | 2 | 2 | 1.36 | 1.36 | 2.72 |
| *Cuscutaceae* | 0 | 1 | 0 | 1 | 0.00 | 0.68 | 0.68 |
| *Scrophulariaceae* | 3 | 2 | 4 | 3 | 2.72 | 2.04 | 4.76 |
| *Lamiaceae* | 5 | 4 | 5 | 6 | 3.40 | 4.08 | 7.48 |
| *Plantaginaceae* | 0 | 1 | 0 | 1 | 0.00 | 0.68 | 0.68 |
| *Chenopodiaceae* | 1 | 1 | 1 | 1 | 0.68 | 0.68 | 1.36 |
| *Polygonaceae* | 2 | 2 | 4 | 6 | 2.72 | 4.08 | 6.80 |
| *Thymelaeaceae* | 1 | 0 | 1 | 0 | 0.68 | 0.00 | 0.68 |
| *Euphorbiaceae* | 1 | 1 | 1 | 1 | 0.68 | 0.68 | 1.36 |
| *Urticaceae* | 0 | 1 | 0 | 2 | 0.00 | 1.36 | 1.36 |
| *Betulaceae* | 1 | 1 | 1 | 1 | 0.68 | 0.68 | 1.36 |
| *Salicaceae* | 2 | 1 | 4 | 2 | 2.72 | 1.36 | 4.08 |
| *Orchidaceae* | 4 | 2 | 5 | 2 | 3.40 | 1.36 | 4.76 |
| *Alliaceae* | 1 | 0 | 3 | 0 | 2.04 | 0.00 | 2.04 |
| *Liliaceae* | 4 | 3 | 6 | 4 | 4.08 | 2.72 | 6.80 |
| *Juncaceae* | 0 | 1 | 0 | 2 | 0.00 | 1.36 | 1.36 |
| *Araceae* | 1 | 0 | 3 | 0 | 2.04 | 0.00 | 2.04 |
| *Cyperaceae* | 1 | 0 | 3 | 0 | 2.04 | 0.00 | 2.04 |
| *Poaceae* | 2 | 5 | 5 | 6 | 3.40 | 4.08 | 7.48 |
| **Gymnosperms** | | | | | | | |
| *Ephedraceae* | 1 | 1 | 1 | 2 | 0.68 | 1.36 | 2.04 |
| *Cupressaceae* | 1 | 1 | 3 | 2 | 2.04 | 1.36 | 3.40 |
| *Taxaceae* | 1 | 1 | 1 | 1 | 0.68 | 0.68 | 1.36 |
| *Pinaceae* | 2 | 2 | 2 | 3 | 1.36 | 2.04 | 3.40 |
| **Pteridophytes** | | | | | | | |
| *Osmundaceae* | 1 | 1 | 1 | 1 | 0.68 | 0.68 | 1.36 |
| *Polypodiaceae* | 2 | 3 | 2 | 3 | 1.36 | 2.04 | 3.40 |
| *Cryptogrammaceae* | 1 | 1 | 2 | 1 | 1.36 | 0.68 | 2.04 |
| *Adiantaceae* | 1 | 1 | 1 | 1 | 0.68 | 0.68 | 1.36 |
| *Aspleniaceae* | 0 | 1 | 0 | 2 | 0.00 | 1.36 | 1.36 |
| *Athyriaceae* | 0 | 2 | 0 | 2 | 0.00 | 1.36 | 1.36 |
| *Dryopteridaceae* | 0 | 1 | 0 | 3 | 0.00 | 2.04 | 2.04 |
|  |  |  |  |  | **100** | **100** |  |

***BG site= Bhundar-Ghangaria site; TLR site = Tolma-Lata-Raini site**
